# Intrahepatic cholangiocarcinoma mimicking a hypervascular mesenchymal tumor: a case report

**DOI:** 10.1093/jscr/rjag338

**Published:** 2026-04-30

**Authors:** Weiwei Wang, Weibo Wen, Ze Liang, Qingmei Pei, Jianguo Pei

**Affiliations:** Department of Nuclear Medicine, Yanbian University Hospital, Yanji 133000, Jilin, China; Department of Nuclear Medicine, Yanbian University Hospital, Yanji 133000, Jilin, China; Department of Nuclear Medicine, Tianjin Nankai Hospital, Tianjin Medical University, Tianjin 300100, China; Department of Radiology, Tianjin Nankai Hospital, Tianjin Medical University, Tianjin 300100, China; Department of Nuclear Medicine, Yanbian University Hospital, Yanji 133000, Jilin, China

**Keywords:** intrahepatic cholangiocarcinoma, multifocal liver occupying lesions, tumor markers were negative, case report

## Abstract

This article reports a case of multifocal intrahepatic cholangiocarcinoma (iCCA) with imaging findings that are easily misdiagnosed and serological tests are completely negative and reveal its key diagnostic pitfalls. The patient was a 54-year-old female who presented with abdominal discomfort. Serum tumor markers were normal. Enhanced computed tomography showed marginal enhancement in arterial phase, slow centripetal filling in delayed phase, and target sign (high signal and low signal ring) in hepatobiliary phase of magnetic resonance imaging. iCCA was finally diagnosed by pathology. This case emphasizes that even with negative tumor markers, iCCA should be considered in patients with atypical imaging manifestations, especially with "target-like" signs. Early pathological biopsy is the key to avoid misdiagnosis and ensure accurate treatment.

## Introduction

Intrahepatic cholangiocarcinoma (iCCA) is one of the prevalent types of primary liver cancer, and its incidence ranks second only to hepatocellular carcinoma [1]. Imaging examinations play a vital role in the diagnosis of liver space-occupying lesions [2]. Nevertheless, the imaging manifestations of iCCA exhibit a high degree of heterogeneity [3]. Particularly when they present nontypical characteristics, they can readily lead to misdiagnosis. Although serum tumor markers are routinely employed for screening, a negative result does not entirely exclude the possibility of malignancy [3]. We present a case of multifocal iCCA with completely negative serum tumor markers and highly misleading imaging results, aiming to expose the diagnostic pitfalls of such cases and emphasize the crucial role of early pathological biopsy in preventing misdiagnosis.

## Case report

A 54-year-old female, complained of stomach discomfort and came to our hospital. CEA, AFP, and CA19-9 were among the assessed tumor markers in the serum, all of which were within normal levels, creating an incorrect early impression that there was a low probability of malignancy.

**Figure 1:**
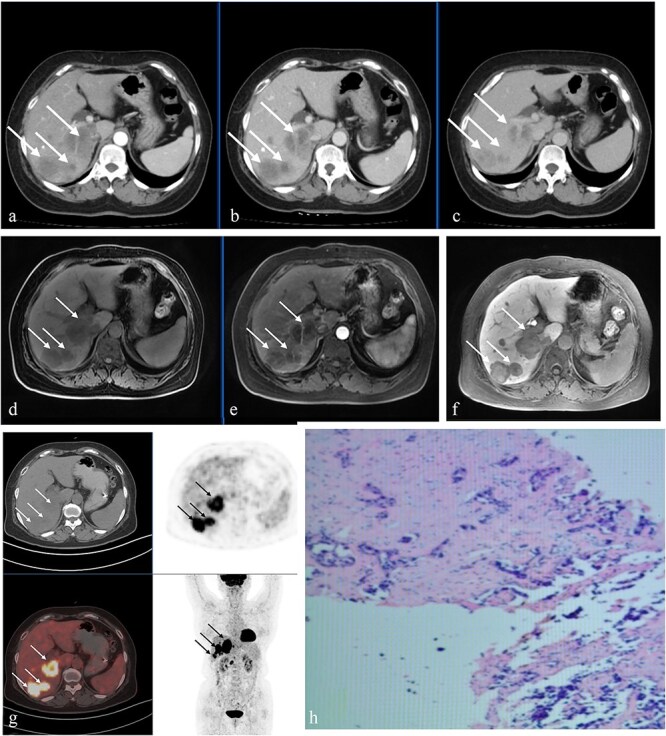
(a–c) Contrast-enhanced upper abdominal CT showing multiple enhanced hepatic nodules and masses (white arrows). (d–f) Contrast-enhanced upper abdominal MRI showing peripheral rim arterial enhancement with central nonenhancement (d), progressive centripetal fill-in on the portal venous phase (e), and central hyperintensity with a peripheral hypointense rim (f) on the hepatobiliary phase (white arrows). (g) PET/CT suggests multiple hypermetabolic hepatic lesions (white/black arrows) and hypermetabolic retroperitoneal lymph nodes. (h) Histopathology shows adenocarcinoma with necrosis.

Numerous hepatic masses of varying sizes scattered throughout the liver were visible on cross-sectional imaging. We observed a distinguishing pattern on contrast-enhanced computed tomography (CT): the lesions showed smooth, uniform peripheral rim augmentation in the arterial stage (Fig. 1a), but contrast moved gradually and only partially toward the center in the delayed and portal venous stages (Fig. 1b and c). This created an image of a minor “seeping” rather than a complete homogeneous filling. Even more complexity was brought about by gadoxetate-enhanced magnetic resonance imaging (MRI) (Fig. 1d and e). Lesions in the hepatobiliary stage (Fig. 1f) showed a remarkable target-like outline with a bright center flanked by a prominent dark rim. This imaging hallmark is more frequently linked to hypervascular mesenchymal malignancies. The lesions suggested adjacent lymph nodes with high metabolic activity and strong ring-shaped uptake (SUVmax 12.7), which indicated a potent biological mechanism (Fig. 1g).

Collectively, the distinctive hypervascular enhancement pattern, the multiple areas of involvement, standard tumor markers, and the hepatobiliary stage showing a false target-like form resulted in a radiologic image closer to being associated with epithelioid hemangioendothelioma [4] or hepatic angiosarcoma [5], instead of iCCA [6]. Comparable hepatobiliary target characteristics are uncommon in iCCA [6] but have been reported in infrequent hypervascular liver tumors [7, 8], which elucidates the diagnostic challenge. These imaging results might readily enable physicians to make inaccurate diagnoses or treatment decisions in the absence of confirmed tissues, delaying appropriate intervention.

A percutaneous needle biopsy guided by ultrasonography provided a conclusive diagnosis. A mildly differentiated adenocarcinoma was identified by histopathology (Fig. 1h). Whereas CDX-2, TTF-1, CA19-9, and S100P yielded negative results, thus eliminating other key sources, immunohistochemical staining indicated high CK19 and CK7 positivity, which corroborated a biliary epithelial origin. In stark contrast to the original ideas based solely on imaging, iCCA was confirmed as the definitive diagnosis by the immunoprofile. Systemic therapy, which suited the patient’s clinical status, was initiated after multidisciplinary discussion.

## Discussion

iCCA constitutes the second most ubiquitous form of primary hepatic malignancy [3]. Nevertheless, its radiological manifestations demonstrate profound heterogeneity, especially when displaying nonclassical features, culminating in a substantial rate of erroneous diagnosis. Per published data, fewer than 5% of iCCA instances manifest a targetoid pattern during the hepatobiliary phase of MRI [9, 10]. This finding is more frequently encountered in hypervascular mesenchymal neoplasms, namely epithelioid hemangioendothelioma [4] and angiosarcoma [5].

In the present case, the imaging appraisal diverged markedly from the accurate interpretation, attributable to the coexistence of a multifocal hepatic lesion exhibiting arterial-phase peripheral enhancement, gradual inward opacification during the delayed phase, a classic bull’s-eye configuration in the hepatobiliary phase, and entirely unremarkable serum tumor markers.

The present case highlights several vital points. When viewed without pathological evidence, particularly characteristic imaging findings may be deceptive, and typical tumor markers may unintentionally reinforce false reassurance. iCCA poses a serious diagnostic risk owing to its wide imaging variability, specifically when it resembles hypervascular mesenchymal tumors. Notwithstanding the results of tumor markers, clinicians should explore early biopsy for multifocal liver lesions that exhibit contradicting or unique imaging properties to evade misdiagnosis and ensure timely, applicable treatment.
